# Reduced gut microbiome protects from alcohol-induced neuroinflammation and alters intestinal and brain inflammasome expression

**DOI:** 10.1186/s12974-018-1328-9

**Published:** 2018-10-27

**Authors:** Patrick P. Lowe, Benedek Gyongyosi, Abhishek Satishchandran, Arvin Iracheta-Vellve, Yeonhee Cho, Aditya Ambade, Gyongyi Szabo

**Affiliations:** Department of Medicine, University of Massachusetts Medical School, 364 Plantation Street, Worcester, MA 01605 USA

**Keywords:** Neuroinflammation, Alcohol, Microglia, Cytokines, Microbiome, Inflammasome

## Abstract

**Background:**

The end-organ effects of alcohol span throughout the entire body, from the gastrointestinal tract to the central nervous system (CNS). In the intestine, alcohol use changes the microbiome composition and increases gut permeability allowing translocation of microbial components into the circulation. Gut-derived pathogen-associated signals initiate inflammatory responses in the liver and possibly elsewhere in the body. Because previous studies showed that the gut microbiome contributes to alcohol-induced liver disease, we hypothesized that antibiotic administration to reduce the gut microbiome would attenuate alcohol-induced inflammation in the brain and small intestine (SI).

**Methods:**

Six- to 8-week-old C57BL/6J female mice were fed alcohol in a liquid diet or a calorie-matched control diet for 10 days with an acute alcohol binge or sugar on the final day (acute-on-chronic alcohol administration). Some mice were treated with oral antibiotics daily to diminish the gut microbiome. We compared serum levels of TNFα, IL-6, and IL-1β by ELISA; expression of cytokines *Tnfα*, *Mcp1*, *Hmgb1*, *Il-17*, *Il-23*, *Il-6*, and *Cox2*; and inflammasome components *Il-1β*, *Il-18*, *Casp1*, *Asc*, and *Nlrp3* in the CNS and SI by qRT-PCR. Microglial morphology was analyzed using immunohistochemical IBA1 staining in the cortex and hippocampus.

**Results:**

Antibiotics dramatically reduced the gut microbiome load in both alcohol- and pair-fed mice. Alcohol-induced neuroinflammation and increase in SI cytokine expression were attenuated in mice with antibiotic treatment. Acute-on-chronic alcohol did not induce serum TNFα, IL-6, and IL-1β. Alcohol feeding significantly increased the expression of proinflammatory cytokines such as *Tnfα*, *Mcp1*, *Hmgb1*, *Il-17*, and *Il-23* in the brain and intestine. Reduction in the gut bacterial load, as a result of antibiotic treatment, attenuated the expression of all of these alcohol-induced proinflammatory cytokines in both the brain and SI. Alcohol feeding resulted in microglia activation and morphologic changes in the cortex and hippocampus characterized by a reactive phenotype. These alcohol-induced changes were abrogated following an antibiotic-induced reduction in the gut microbiome. Unexpectedly, antibiotic treatment increased the mRNA expression of some inflammasome components in both the brain and intestine.

**Conclusions:**

Our data show for the first time that the acute-on-chronic alcohol administration in mice induces both neuroinflammation and intestinal inflammation and that reduction in the intestinal bacterial load can attenuate alcohol-associated CNS and gut inflammation. Gut microbiome-derived signals contribute to neuroinflammation in acute-on-chronic alcohol exposure.

## Background

Prolonged alcohol consumption leads to translocation of gut bacterial components, such as endotoxin, from the intestinal lumen into the circulation [1–3]. Once absorbed, alcohol along with gut-derived endotoxin is delivered via the portal circulation to the liver where metabolism begins and an inflammatory cascade is initiated. However, endotoxin, unmetabolized alcohol, and alcohol metabolites also pass through the liver and reach the systemic circulation and other organs, including the peripheral immune system and the central nervous system (CNS). While previous studies have investigated the direct effects of alcohol on the brain [4–6], little is known about the role of gut-derived microbial products and their impact on the nervous system and neuroinflammation.

Microglia play a critical role in sensing and responding to alcohol consumption and are involved in multiple immune signaling pathways [7–10]. Microglia express Toll-like receptor 4 (TLR4), a pattern recognition receptor critical in alcohol-induced neuroinflammation [11–13] as well as the NLR family pyrin domain containing 3 (NLRP3) inflammasome [9]. Previous studies showed that TLR4 knockout mice are protected from increased cytokine expression in various regions of the brain and from increased activation of microglia [14–16]. TLR4 recognizes endogenous danger signals such as HMGB1 [17, 18] and is the major pattern recognition receptor of bacterial endotoxin (also known as lipopolysaccharide (LPS)) [19]. Although endotoxin is not generally believed to cross the blood-brain barrier [20], data from TLR4 knockout mice suggests that signaling through TLR4 is an important component influencing alcohol-induced neuroinflammation. Neuroinflammation is mediated by the inflammasome complex, a multiprotein complex that senses pathogens and danger signals leading to cleavage and release of proinflammatory IL-1β and IL-18 [9].

LPS signaling is also a critical component of liver pathology associated with alcohol consumption. Alcohol metabolism leads to cell stress, hepatocyte damage, and release of sterile danger signals in the liver [21, 22]. Endotoxins, derived from the intestinal microbiome into the portal circulation, are recognized by pattern recognition receptors such as TLR4 and initiate an inflammatory response secondary to the hepatocyte stress and damage caused by the release of reactive oxygen species and other cellular stresses induced by alcohol metabolism. Interestingly, we and others have shown that treating mice with antibiotics to reduce the bacterial load in the gastrointestinal tract (and thereby reducing endotoxin levels) attenuates liver inflammation and steatosis after alcohol use [23–25]. This reduction in gut bacterial load could ameliorate the alcohol-induced changes in the brain.

To further explore the critical role of the gut microbiome in the gut-brain axis, we used antibiotics to reduce the intestinal bacterial load in mice. Following acute-on-chronic alcohol consumption in mice (10 days of alcohol followed by an acute alcohol binge), we show that alcohol induces neuroinflammation in the CNS and also increases cytokine expression in the small intestine. Inflammation in both organs was attenuated with antibiotic-induced microbiome reduction. Interestingly, although cytokine expression was reduced, antibiotic treatment induced the mRNA expression of inflammasome components and cytokines processed by the inflammasome in the CNS and intestine. These results show for the first time that manipulation of the gut microbiome via reduction of the microbial load protects from alcohol-induced CNS and intestinal inflammation. Our study provides important insights into the interactions of the intestinal microbiome and brain in the gut-brain axis induced by alcohol.

## Methods

### Mouse alcohol feeding

All animal studies were approved by the Institutional Animal Care and Use Committee at the University of Massachusetts Medical School (UMMS). Wild-type C57BL/6J 6- to 8-week-old female mice were purchased from Jackson Laboratories and co-housed in the UMMS Animal Medicine Facility. Female mice were chosen because they are more susceptible to alcohol-induced liver injury than male mice [26–28]. Alcohol feeding followed the acute-on-chronic model previously described by Bertola et al. [29]. Briefly, all mice received the pair-fed Lieber-DeCarli (Bio-Serv) liquid diet for 5 days. Some mice then received 5% alcohol and maltose dextran in a liquid diet while pair-fed mice remained on the control liquid diet. Pair-fed mice were calorie-matched with the alcohol-fed mice. Nine hours prior to sacrifice, alcohol-fed mice received alcohol via oral gavage (5 g kg^−1^ body weight) and pair-fed mice received isocaloric maltose dextran.

### Antibiotic treatment

Mice were either treated twice daily with an oral intragastric gavage of water or a broad spectrum antibiotic cocktail (Abx) containing ampicillin (100 mg/kg body weight (BW); Sigma), neomycin (100 mg/kg BW; Gibco), metronidazole (100 mg/kg BW; Sigma), and vancomycin (50 mg/kg BW; Sigma). Gavages began on the first day of liquid diet and continued daily until the completion of alcohol feeding. Significant reduction in bacterial load was confirmed by bacterial culture (described below) similar to previous reports [23].

### Bacterial culture

Mouse feces were collected directly from the anus and suspended in thioglycolate media. Suspensions were plated on non-selective LB agar plates (EMD Millipore) and incubated for 24 h at 37 °C for assessment of bacterial load reduction.

### qPCR analysis

RNA extraction from the small intestine and brain cortical tissue was performed using miRNeasy Extraction Kit (Qiagen) according to the manufacturer’s instructions, including on-column DNase digestion (Zymo Research). Reverse transcription for cDNA was completed from 1 μg of RNA and subsequent 1:5 dilution in nuclease-free water. Real-time qPCR using SYBR Green (BioRad) was performed according to the manufacturer’s instructions. RT-qPCR primers are listed in Table 1, and *18S* mRNA expression was used as a housekeeping gene for 2^−ΔΔ*Ct*^ method of RNA expression analysis. For 16S comparison between antibiotic-treated and non-treated animals, stool bacterial DNA was extracted using QIAamp DNA Stool Mini Kit (Qiagen) according to the manufacturer’s protocol. After running a qPCR reaction using 16S primers similar to described above, a Δ*Ct* was calculated using the average *Ct* value of each sample duplicate and subtracting the average Δ*Ct* of untreated pair-fed mice. The bacterial 16S PCR product was run on a 1% agarose gel to visualize the relative reduction in bacterial load.

**Table 1:** Real-time PCR primers

Primer	Forward (5′–3′)	Reverse (5′–3′)
*18S*	GTAACCCGTTGAACCCCATT	CCATCCAATCGGTAGTAGCG
*16S*	TCCTACGGGAGGCAGCAGT	GGACTACCAGGGTATCTAATCCTGTT
*Tnfα*	GAAGTTCCCAAATGGCCTCC	GTGAGGGTCTGGGCCATAGA
*Mcp-1*	CAG GTC CCT GTC ATG CTT CT	TCTGGACCCATTCCTTCTTG
*Il-1β*	TCTTTGAAGTTGACGGACCC	TGAGTGATACTGCCTGCCTG
*Il-17*	CAGGGAGAGCTTCATCTGTGT	GCTGAGCTTTGAGGGATGAT
*Il-23*	AAGTTCTCTCCTCTTCCCTGTCGC	TCTTGTGGAGCAGCAGATGTGAG
*Hmgb1*	CGCGGAGGAAAATCAACTAA	TCATAACGAGCCTTGTCAGC
*Il-6*	ACAACCACGGCCTTCCCTACTT	CACGATTTCCCAGAGAACATGTG
*Cox2*	AACCGAGTCGTTCTGCCAAT	CTAGGGAGGGGACTGCTCAT
*Nlrp3*	AGCCTTCCAGGATCCTCTTC	CTTGGGCAGCAGTTTCTTTC
*Asc*	GAAGCTGCTGACAGTGCAAC	GCCACAGCTCCAGACTCTTC
*Casp1*	AGATGGCACATTTCCAGGAC	GATCCTCCAGCAGCAACTTC
*Il-18*	CAGGCCTGACATCTTCTGCAA	TCTGACATGGCAGCCATTGT

The above forward and reverse sequences of primers were used in real-time PCR

*Abbreviations*: *Tnfα* tumor necrosis factor-α, *Mcp-1* monocyte chemoattractant protein 1 (encoded by *CCL2*), *Il-1β* interleukin-1β, *Il-17* interleukin-17, *Il-23* interleukin-23, *Hmgb1* high-mobility group box 1, *Il-6* interleukin 6, *Cox2* cyclooxygenase 2, *Nlrp3* NLR family pyrin domain containing 3, *Asc* apoptosis-associated speck-like protein (encoded by *PYCARD*), *Casp1* caspase-1 (encoded by *CASP1*), *Il-18* interleukin-18

### Serum cytokine measurement

Mice were cheek-bled prior to sacrifice, and serum was isolated. TNFα and IL-6 (Biolegend, San Diego, CA, USA) and IL-1β (R&D Systems, Minneapolis, MN, USA) were measured by ELISA.

### Immunohistochemistry

Following sacrifice, brain tissue was dissected and fixed in 10% formalin overnight before paraffin embedding. Immunohistochemical staining was completed at the UMMS Morphology Core using anti-ionized calcium-binding adapter molecule (IBA1) antibody (Wako; 1:1000) and subsequently labeled with streptavidin-biotin immunoenzymatic antigen for detection with 3,3′-diaminobenzidine (DAB) (UltraVision Mouse Tissue Detection System Anti-Mouse HRP/DAB; Lab Vision). Images were acquired from the described CNS areas by light microscopy (cortex; CA1, CA3, and DG of the hippocampus) at × 40 magnification for process length and cell body size measurements of microglia using ImageJ. Cell process length for each microglial cell was measured by tracing all extensions off of the soma to their distal termination using ImageJ’s freehand measuring tool. For each microglia, the length of all processes was summed to obtain the total cell process length. The soma area was measured by tracing the perimeter of the cell body and measuring the contained area using ImageJ’s freehand tracer and the area measurement function. Microglia were analyzed from five to nine images taken randomly from each CNS region from each mouse. The investigator was blinded to the sample groups during staining, image acquisition, and ImageJ analysis. IBA1 positivity was measured using the *Color Deconvolution* plug-in in ImageJ.

### Statistical analysis

Statistical analysis was carried out using GraphPad Prism Version 7.0 using Mann-Whitney test. *p* < 0.05 was considered statistically significant. Outlier exclusion was calculated using Grubbs’ outlier test with alpha set to 0.05.

## Results

### Antibiotic treatment dramatically decontaminates gut bacterial load

**Fig. 1:**
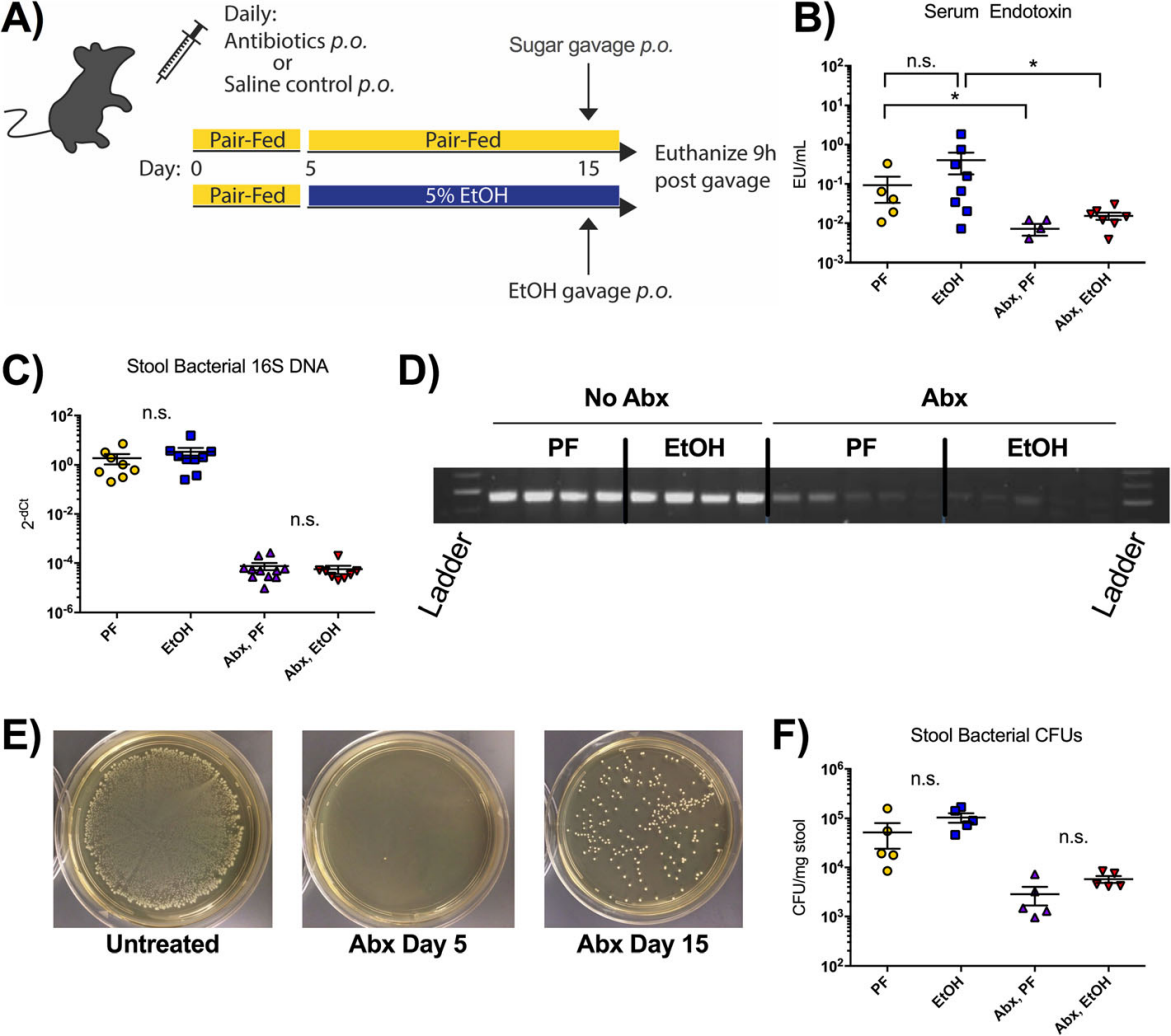
Oral antibiotics significantly reduce the gut bacterial load. **a** Four groups of wild-type C57BL/6J female mice were treated with pair-fed diet (PF; *n* = 5), 5% alcohol diet (EtOH; *n* = 10), oral antibiotics (Abx) with PF (*n* = 6), or Abx with EtOH (*n* = 9). An acute sugar or alcohol binge was given 9 h before sacrifice. **b** Serum endotoxin was measured at sacrifice to determine translocation of gut bacterial products into systemic circulation. **c** DNA was isolated from the stool of PF and EtOH mice before sacrifice, and 16S DNA was measured by qPCR using universal 16S primers. **d** The PCR products from **c** were run on an agarose gel for a general comparison of the four groups. **e** Stools were resuspended in thioglycolate and plated on non-selective agar to measure gut bacterial load prior to antibiotic treatment (untreated), after 5 days of Abx treatment (Abx day 5), and at the end of the experiment (Abx day 15). **f** Colony-forming units (CFUs) were quantified from stool extracted at sacrifice on day 15. Data are mean ± SEM, *n* = 5–10 mice/group. *p < 0.05; n.s., not significant

While the modulating effects of chronic alcohol administration have been studied in the gut microbiome, alcoholic liver disease, and neuroinflammation, it is unclear how shorter alcohol use and/or alcohol binge affect inflammation signaling in the CNS and what role the gut microbiome plays in this process. In this study, mice received 5% alcohol (EtOH) in a liquid diet for 10 days (after a 5-day liquid diet acclimation period), followed by a one-time alcohol binge or a calorie-matched pair-fed (PF) diet [29]. Female mice were chosen because they have greater sensitivity to alcohol, and previous studies have focused on female animals [26–28]. To elucidate the importance of the gut microbiome in the translocation of pathogen-associated molecular patterns (PAMPs) from the intestine to extra-enteric organs, we used oral administration of a cocktail of antibiotics (ampicillin, neomycin, vancomycin, and metronidazole) to drastically reduce the bacterial load in the gut (Fig. 1a). Oral antibiotic treatment (Abx) caused a significant reduction in endotoxin in the circulation at the time of sacrifice both in pair-fed and alcohol-fed mice (Fig. 1b). The expression of 16S bacterial DNA, measured from mice stools collected immediately prior to sacrifice, was dramatically reduced by antibiotic treatment (Fig. 1c, d). Stool bacteria cultured on non-selective agar plates also revealed almost complete elimination of culturable colonies after 5 days of antibiotic treatment (Fig. 1e). Some recovery of bacteria in the stool was observed by the conclusion of the 15-day study, likely due to the development of antibiotic resistance (Fig.1e). However, bacterial colony-forming units (CFUs) were dramatically reduced in the stool obtained on the day of sacrifice in antibiotic-treated animals compared with untreated mice (Fig.1f). Together, these data indicate that antibiotic treatment successfully suppressed gut bacterial load and reduced circulating endotoxin in both pair- and alcohol-fed mice.

### Gut decontamination abrogates alcohol-induced proinflammatory cytokine expression in the brain cortex

**Fig. 2:**
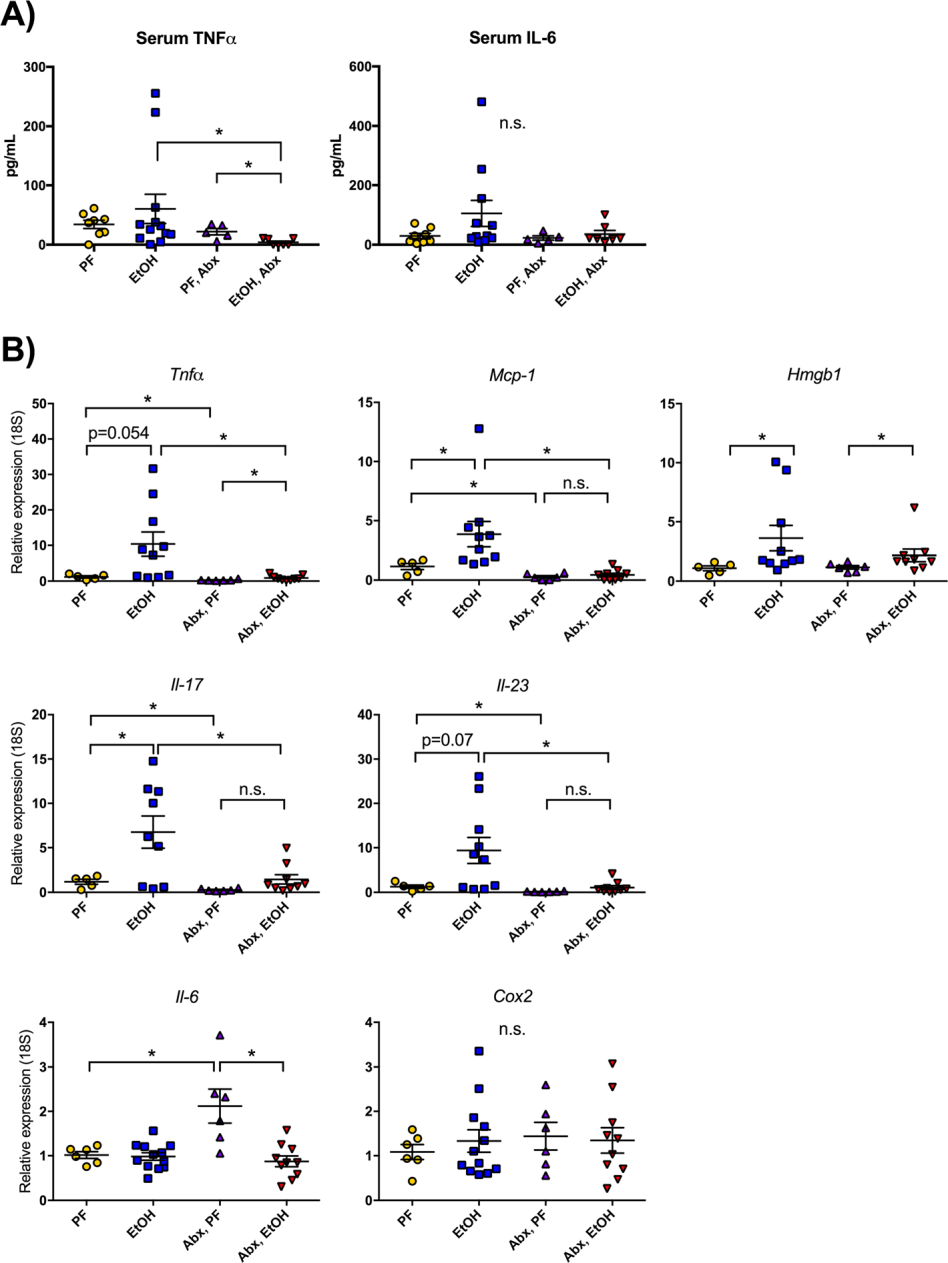
Antibiotic treatment protects from alcohol-induced inflammatory cytokine expression in the cortex. **a** Serum TNFα and IL-6 were measured by ELISA. **b** Expression levels of proinflammatory cytokines *Tnfα*, *Mcp1*, *Hmgb1*, *Il-17*, *Il-23*, *Il-6*, and *Cox2* were measured from the cortex of pair-fed (PF) or alcohol-fed (EtOH) mice with or without daily antibiotic treatment (Abx). Data are mean ± SEM, *n* = 5–10 mice/group. *p < 0.05; n.s., not significant

Chronic alcohol induces circulating proinflammatory cytokines in both animal models and in human patients [30, 31]. To determine whether this systemic cytokine induction also occurs in the acute-on-chronic model in mice, we measured circulating TNFα and IL-6 in the serum (Fig. 2a). While alcohol did not induce statistically significant increases in either cytokine, antibiotic treatment significantly reduced circulating TNFα in both pair-fed and alcohol-fed mice (Fig. 2a).

Chronic alcohol use results in neuroinflammation both in humans and in mice [7, 12]. We found that 10 days of chronic alcohol feeding followed by a one-time binge in mice, a model of acute-on-chronic alcohol consumption not previously used to study neuroinflammation, induced significantly higher expression of proinflammatory cytokine genes including *Mcp-1*, *Hmgb1*, and *Il-17* and non-significant trends toward increased expression of *Tnfα* and *Il-23* in the brain cortex (Fig. 2b). *Tnfα*, *Mcp-1*, *Hmgb1*, *Il-17*, and *Il-23* are proinflammatory cytokines that can be released by multiple cell types, and each has previously been associated with alcohol-induced neuroinflammation [9, 14, 32, 33]. Alcohol did not induce expression of *Il-6* or *Cox2*. Interestingly, *Il-6* was induced in pair-fed antibiotic-treated mice compared to non-treated mice, and alcohol feeding reduced this induction (Fig. 2b).

Previous studies indicate that antibiotic treatment that reduces intestinal bacterial load also reduces alcohol-induced inflammation in the liver [23]. Here, we hypothesized that translocation of gut bacterial products to the CNS contributes to alcohol-induced neuroinflammation and that this process is regulated by the gut microbial load. Therefore, we sought to investigate whether gut decontamination could protect from neuroinflammation associated with alcohol consumption. We observed that the proinflammatory cytokine expression increase in the cortex in alcohol-fed mice compared to PF controls was markedly reduced in mice treated with Abx (Fig. 2b). Antibiotic treatment fully prevented alcohol-related induction of *Mcp1*, *Il-17*, and *Il-23* mRNA expression in the cortex. *Tnfα* was induced in antibiotic-treated, alcohol-fed mice compared to antibiotic-treated pair-fed mice, but its expression was still significantly lower compared to alcohol-fed mice without antibiotic treatment. Expression of *Tnfα*, *Mcp1*, *Il-17*, and *Il-23* was also reduced in the cortex of antibiotic-treated pair-fed mice compared to those without antibiotic treatment. These results indicate that acute-on-chronic alcohol feeding in mice increases proinflammatory cytokine induction that is prevented by the reduction in gut-derived PAMPs and the gut microbiome.

### Cortical expression of inflammasome components increase with bacterial decontamination

**Fig. 3:**
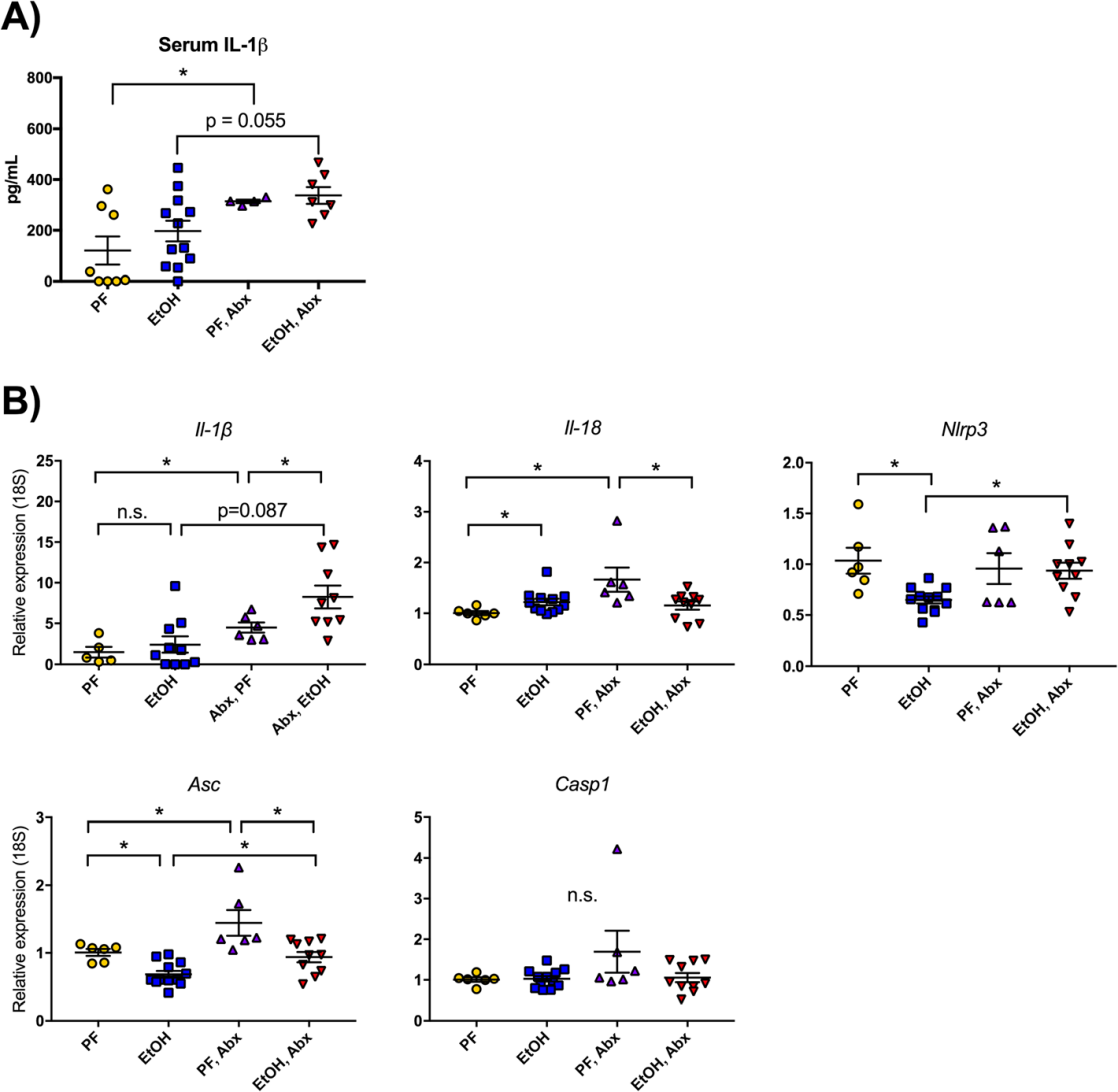
The expression levels of inflammasome components and *Il-1β* are increased in the cortex after antibiotic decontamination. **a** Serum IL-1β was measured by ELISA. **b** Cortical expression of the inflammasome components *Nlrp3*, *Asc*, and *Casp1* as well as the cytokines *Il-1β* and *Il-18* were measured from the brains of pair-fed (PF) or alcohol-fed (EtOH) mice with or without daily antibiotic treatment (Abx). Data are mean ± SEM, *n* = 5–10 mice/group. *p < 0.05

Because we found that multiple proinflammatory cytokines were reduced in the cortex of antibiotic-treated mice (Fig. 2), we next measured inflammasome-related transcripts to elucidate if alcohol or antibiotics influenced inflammasome-mediated cytokine expression. The inflammasome is a multiprotein complex containing NOD-like receptors (NLRs, including NLRP3) that can sense pathogens and danger signals, the adaptor molecule, ASC, and the effector molecule, caspase-1. Inflammasome activation leads to cleavage of pro-IL-1β and pro-IL-18 to their respective bioactive forms, IL-1β and IL-18 [9]. We found that although alcohol did not induce IL-1β, antibiotic treatment increased circulating serum IL-1β in pair-fed mice (*p* < 0.05) and trended toward an increase in alcohol-fed mice (*p* = 0.055) (Fig. 3a). Interestingly, although chronic alcohol consumption models have led to increased expression of inflammasome components and *Il-1β* [9], we found no significant increase in alcohol-induced *Il-1β* mRNA expression in this acute-on-chronic alcohol model (Fig. 3b). However, cortical *Il-1β* mRNA expression in antibiotic-treated pair-fed mice was significantly increased and we observed an increasing trend in *Il-1β* in antibiotic-treated alcohol-fed compared to untreated mice. Interestingly, in antibiotic-treated mice, alcohol administration significantly increased *Il-1β* mRNA expression compared to pair-fed mice. Expression of *Il-18* was induced in alcohol-fed mice in the cortex, and similar to the increase in pair-fed *Il-1β*, we also found that *Il-18* and *Asc* were elevated in antibiotic-treated, PF mice compared to untreated PF mice (Fig. 3b). Acute-on-chronic alcohol administration reduced the expression of *Nlrp3* and *Asc* and increased the expression of *Il-18* in untreated alcohol-fed mice compared to untreated PF controls. *Asc* and *Il-18* mRNA expression were reduced in antibiotic-treated compared to untreated alcohol-fed mice (Fig. 3b). *Caspase-1* mRNA levels did not change significantly in any of the treatment groups (Fig. 3b). These observations suggest that regulation of the inflammasome and IL-1β depends on the gut microbiome and is minimally influenced in the acute-on-chronic alcohol model in mice.

### Gut decontamination alters cortical and hippocampal microglia

**Fig. 4:**
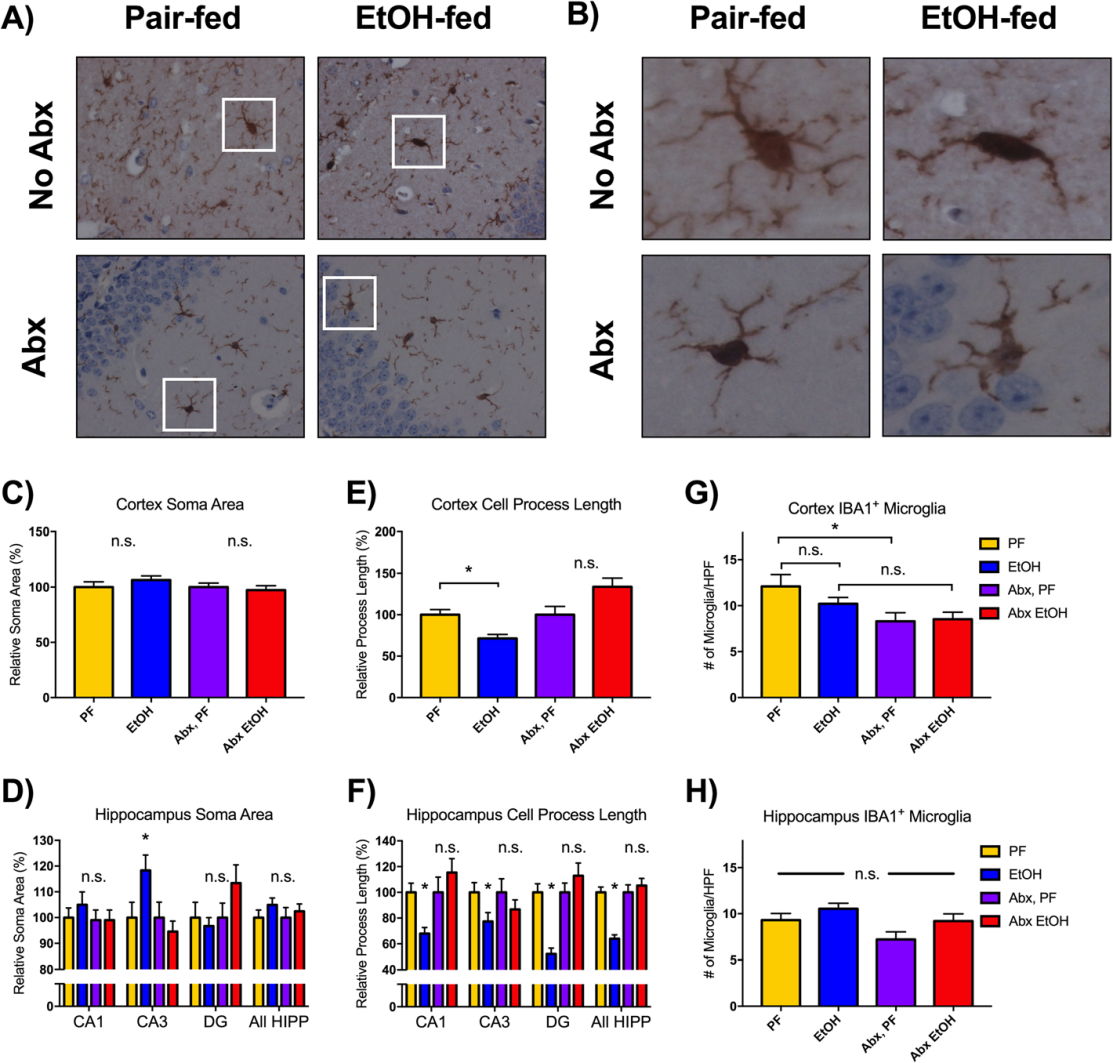
Antibiotic treatment prevents alcohol-induced morphological changes in cortical and hippocampal microglia. **a** Microglia were immunohistochemically stained for IBA1 and visualized at × 40 magnification in the cortex of pair-fed (PF) or alcohol-fed (EtOH) mice. Representative microglia from the insets are shown in **b**. **c**–**d** For both the cortex and hippocampus, microglial soma area was measured by tracing the perimeter of the cell body and calculating the area. **e**–**f** Cell process length was measured in cortical and hippocampal microglia by summing the length of all extensions off of the soma to their distal termination and normalized to the respective PF controls. IBA1-positive staining microglia were quantified in the cortex (**g**) and hippocampus (**h**). Data are mean ± SEM, *n* = 3 mice/group and 5–9 images/region. *p < 0.05

To characterize the effects of the acute-on-chronic alcohol model in the CNS, we next examined the microglia activation. Microglia are the resident macrophages of the CNS capable of expressing proinflammatory cytokines in response to an insult, such as alcohol [34]. Activated microglia are characterized by altered cell morphology, taking on an amoeboid shape with enlarged cell bodies (soma) and shortened peripheral processes [35]. We used immunohistochemistry to identify IBA1-positive microglia (representative images shown in Fig. 4a, b). The soma size and length of cell extensions off the soma were measured in the cortical and hippocampal microglia in all treatment groups and normalized to PF mice. No significant differences in soma size were observed in the cortex (Fig. 4c). Investigation of the sub-regions of the hippocampus, such as the CA1, CA3, and dentate gyrus (DG) areas, revealed that alcohol increased the soma area only in the microglia of the CA3 region. There was no change in the soma area for CA3 microglia in EtOH-fed mice compared to PF controls that were both treated with antibiotics (Fig. 4d). Importantly, we found that alcohol reduced the total process length compared to pair-fed mice in the cortex (Fig. 4e), consistent with the condensed cell morphology characteristic of microglial activation [35]. Antibiotic treatment eliminated this alcohol-induced reduction in process length in cortical microglia. Hippocampal microglia process length in alcohol-fed mice was significantly reduced compared to pair-fed controls in all regions investigated, and as in the cortex, antibiotic treatment eliminated this morphological change (Fig. 4f). The number of microglia in the cortex was not changed in EtOH-fed compared to PF mice in either treatment group, although antibiotic treatment in PF mice modestly reduced the number of cortical microglia compared to untreated PF mice (Fig. 4g). There was no change in microglial numbers in the hippocampus (Fig. 4h).

### Alcohol-induced cytokine expression in the small intestine is attenuated by antibiotic administration

**Fig. 5:**
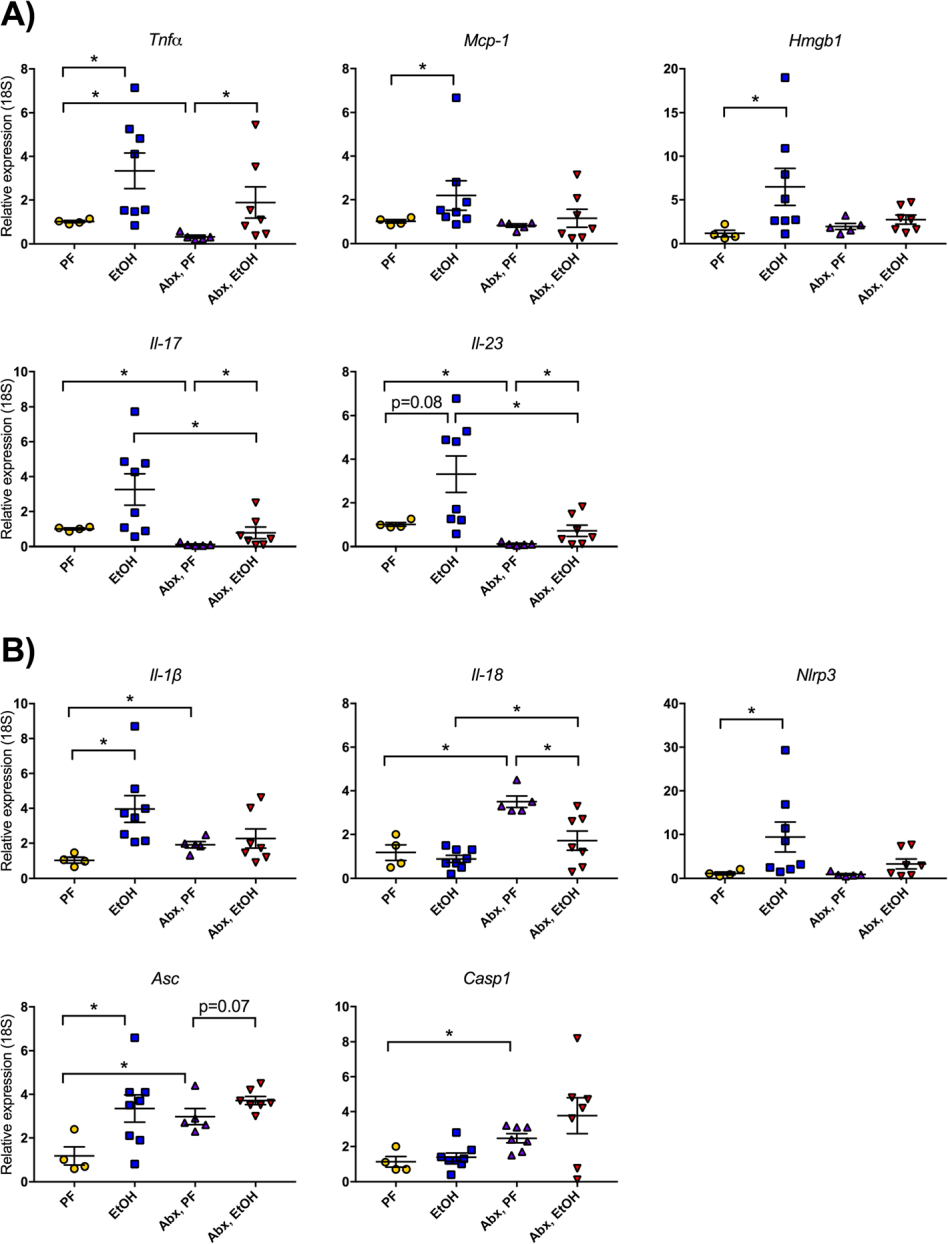
Alcohol-induced small intestinal inflammation is reduced with gut bacterial load reduction. **a** Expression of proinflammatory cytokines *Tnfα*, *Mcp1*, *Hmgb1*, *Il-17*, and *Il-23* was measured from the small intestine of pair-fed (PF) or alcohol-fed (EtOH) mice with or without daily antibiotic treatment (Abx). **b** Expression of inflammasome components *Nlrp3*, *Asc*, and *Casp1* as well as the cytokines *Il-1β* and *Il-18* were measured by qPCR. Data are mean ± SEM, *n* = 5–10 mice/group. *p < 0.05

The alcohol-induced changes we observed in the brain could be due to a loss of integrity of the gut barrier. Previous studies have shown that intestinal cytokine expression can reduce gut barrier integrity and may allow leakage of pathogen-associated molecules from the intestinal lumen into the systemic circulation [36]. Therefore, we measured the intestinal expression of various proinflammatory cytokines and found that they were increased after the acute-on-chronic alcohol administration compared to calorie-matched pair-fed mice (Fig. 5a). The expression of *Tnfα*, *Mcp1*, and *Hmgb1* mRNA was significantly increased in the small intestine following alcohol consumption, and *Il-17* and *Il-23* expression also showed an increasing trend in EtOH mice. Treatment with the antibiotic cocktail reduced the bacterial load in the intestine (Fig. 1) and led to significantly attenuated alcohol-induced *Mcp1* and *Hmgb1* mRNA levels. Antibiotic treatment reduced the baseline expression of the inflammatory cytokines including *Tnfα*, *Il-17*, and *Il-23* in PF mice compared to untreated PF mice (Fig. 5a). Interestingly, even with antibiotic treatment, alcohol feeding still increased the expression of *Tnfα*, *Il-17*, and *Il-23* in the small intestine of antibiotic-treated alcohol-fed mice compared to antibiotic-treated pair-fed mice (Fig. 5a).

Recent research has highlighted an important connection between the intestinal microbiome and inflammasomes [37], particularly the NLRP3 inflammasome [38]. Therefore, we investigated whether antibiotic decontamination of the gut impacted the expression of inflammasome components in the small intestine. Alcohol induced expression of *Il-1β*, *Nlrp3*, and *Asc* compared to pair-fed controls (Fig. 5b). Antibiotic treatment abrogated the alcohol induction of *Il-1β*, *Nlrp3*, and *Asc*, and antibiotics also increased the baseline expression in pair-fed mice of *Il-1β*, *Il-18*, *Asc*, and *Casp1* (Fig. 5b).

## Discussion

In this study, we show that acute-on-chronic alcohol administration results in the central nervous system and small intestinal inflammation and that reducing the gut microbial load with antibiotics protects against alcohol-induced neuroinflammation. The cocktail of oral antibiotics dramatically reduced the gut bacterial load and circulating endotoxin levels. Alcohol-induced neuroinflammation, including microglial morphologic changes and proinflammatory gene expression, was significantly attenuated in oral antibiotic-treated mice, providing novel evidence for the importance of gut bacterial load and PAMPs in the gut-brain axis in alcohol use. We also describe increased proinflammatory cytokine expression in the small intestine after alcohol consumption that can be reduced by treatment with intragastric antibiotics that drastically reduced the bacterial load in the intestine. Interestingly, reduction in the gut microbiome was associated with increased expression of inflammasome components in both the CNS and intestine.

Previously, we have shown that antibiotic treatment in the acute-on-chronic alcohol model protects the liver from alcohol-induced inflammation (including cytokine expression), immune cell infiltration, and steatosis [23]. In the present study, we found evidence of microglial activation by acute-on-chronic alcohol administration in mice. CNS proinflammatory cytokine expression was increased, and average cell process length was decreased in EtOH mice indicating microglia activation. Activated microglia take on an amoeboid-like morphology with reduced process length and, typically, an increased soma size [35]. Acute-on-chronic alcohol reduced cell process length in both the cortex and hippocampus and significantly increased the soma size in part of the hippocampus. Interestingly, although acute-on-chronic alcohol-induced proinflammatory cytokine expression in the CNS, alcohol feeding did not increase circulating levels of TNFα, IL-6, and IL-1β. This indicates that alcohol-induced neuroinflammation may occur independent of systemic inflammation, although further investigation of other peripheral signals will be necessary to rule out contributions from circulating factors.

Similar to observations in the liver [23], antibiotic gut decontamination protected the CNS from proinflammatory gene expression and changes in the resident macrophage population. Interestingly, germ-free mice do not show the same protection from alcohol-induced liver damage that we have previously described using antibiotic decontamination [39]. A possible explanation for these different observations is that some baseline bacterial load and/or presence of bacteria during development is critical for the alcohol-induced response of the immune system as well as for organ-specific immunity. Indeed, previous research has highlighted a role for antibiotic treatment during development in affecting the function of adaptive immune cells [40]. Although multiple studies demonstrated alcohol-induced neuroinflammation after chronic, prolonged alcohol administration in mice and rats, here, we show that a 10-day alcohol feeding followed by an acute binge also results in alcohol-related neuroinflammation. Furthermore, this NIAAA model of alcohol administration results in common end-organ effects of inflammation on the brain, small intestine and liver.

Our data are consistent with previous studies examining the role of TLR4 signaling in alcohol-related organ pathology. While some have suggested that alcohol may interact directly with TLR4 or affect lipid membrane interactions required for proper TLR4 signal transduction [41, 42], TLR4 also recognizes endogenous (including HMGB1) [17, 18] and exogenous (i.e., bacterial components such as LPS) [19] danger signals. Studies show that TLR4 knockout and knockdown mice are protected from numerous inflammation-related sequelae of alcohol exposure in the liver [43] and in the brain [14–16]. Rather than focusing on TLR4 and its signaling pathway, we used antibiotics to reduce bacterial LPS, one of the prominent ligands of TLR4, and reveal a similar reduction in tissue inflammation from the gut to the brain. Our study adds critical evidence to the understanding of the gut-brain axis that relates multifocal pathology in the body after chronic alcohol exposure.

An important remaining question is whether gut bacteria or their products are primarily responsible for organ damage. A direct link between LPS and organ inflammation is possible; leakage of live or dead bacteria or bacterial-derived products into the systemic circulation has been documented in various alcohol administration settings [1, 2, 44, 45]. These bacterial signals could be directly responsible for inducing inflammation in the gut and in the brain, as well as the associated organ damage. Although LPS does not cross the blood-brain barrier at significant levels [20], it could be interacting with juxta-cerebrovascular cells to transmit an immune signal across the barrier. Evidence of blood-brain barrier disruption in alcohol models and human patients provides another explanation for a possible direct mechanism of LPS-induced neuroinflammation [46]. Alternatively, gut-derived signals, such as LPS, bacterial metabolites, or other undescribed intestinal signals, could lead to a systemic reaction. This reaction could include inflammatory cytokines or activated immune cells in the liver or in the circulation that then induce organ-specific inflammation in the CNS and elsewhere in the body. In the present study, we did not detect alcohol-induced increases in circulating TNFα, IL-6, or IL-1β which suggests that alcohol-induced neuroinflammation can be induced by alcohol in the absence of systemic cytokine increases. Developing models to investigate possible peripheral signaling to the CNS leading to neuroinflammation will be a critical area of further study to explain inter-organ communication after alcohol consumption.

Our data supports previous studies showing that alcohol can induce inflammatory signaling in the intestine. This inflammation may be a key factor in the breakdown of the intestinal barrier integrity and ensuing leakage of bacterial products into the circulation associated with alcohol. Using both in vitro and in vivo models, Al-Sadi et al. have shown that proinflammatory cytokines are capable of reducing tight junctions and gut barrier integrity, leading to breakdown and molecule translocation across the gastrointestinal tract [47–49]. Other mechanisms of alcohol-induced loss of intestinal barrier integrity have been explored and include bacterial dysbiosis [50, 51], luminal homeostasis [45, 52], enterocyte cellular stress, and dysregulation of structural proteins [53]. Furthermore, the relationship between proinflammatory gene expression and gut barrier dysfunction appears to be critical [36, 54], and our data further emphasize the role of alcohol and intestinal bacteria in regulating intestinal cytokine levels.

## Conclusion

Our study shows for the first time that acute-on-chronic alcohol induces neuroinflammation and small intestinal proinflammatory cytokine expression. Reducing the gut bacterial load with oral antibiotics protects mice from proinflammatory cytokine expression in the CNS and small intestine and highlights critical connections between intestinal microbiome and the gut-brain axis following alcohol consumption.

## Abbreviations

Abx: Broad spectrum antibiotic cocktail
*Asc*: Apoptosis-associated speck-like protein;
*Casp1*: Caspase-1
CFUs: Colony-forming units
CNS: Central nervous system
*Cox2*: Cyclooxygenase 2
*Hmgb1*: High-mobility group box 1
*Il-17*: Interleukin-17
*Il-18*: Interleukin-18
*Il-1β*: Interleukin-1β
*Il-23*: Interleukin-23
*Il-6*: Interleukin-6
*Mcp-1*: Monocyte chemoattractant protein 1
*Nlrp3*: NLR family pyrin domain containing 3
PAMPs: Pathogen-associated molecular patterns
SI: Small intestine
TLR4: Toll-like receptor 4
*Tnfα*: Tumor necrosis factor-α
